# Enhanced photocatalytic and photodynamic activity of chitosan and garlic loaded CdO–TiO_2_ hybrid bionanomaterials

**DOI:** 10.1038/s41598-021-00242-5

**Published:** 2021-10-21

**Authors:** K. I. Dhanalekshmi, P. Magesan, M. J. Umapathy, Xiang Zhang, N. Srinivasan, K. Jayamoorthy

**Affiliations:** 1 School of Mechanical Engineering, Beijing Institute of Technology, Beijing, 100 081 China; 2 Department of Chemistry, Bharath Institute of Higher Education and Research, Bharath University, Chennai, 600 073 Tamilnadu India; 3 Department of Chemistry, College of Engineering Guindy, Anna University, Chennai, 600 025 Tamilnadu India; 4 Department of Chemistry, Pachiyappa’s College for Men, Kanchipuram, 631 501 Tamilnadu India; 5 Department of Chemistry, St. Joseph’s College of Engineering, Chennai, 600 119 Tamilnadu India

**Keywords:** Biochemistry, Cancer, Environmental sciences, Chemistry, Nanoscience and technology

## Abstract

Herein, the work addresses the synthesis of biomaterials (chitosan and garlic) loaded CdO–TiO_2_ hybrid nanocomposites for photocatalytic water treatment and photodynamic cancer therapeutic applications that were reported the first time. CdO–TiO_2_ (CT) nanocomposites were synthesized and loaded with the biomaterials such as chitosan and garlic by simple sol–gel method. The nanomaterials were characterized and the photodegradation of three model pollutants, Methylene blue (MB), Methyl orange (MO) and Rhodamine B (Rh-B) was opted to investigate the efficiency of the synthesized photocatalyst under the solar light. From the results, the garlic-loaded CdO–TiO_2_ (AS-CT) hybrid nanocomposites exhibit a superior photocatalytic activity than the chitosan-loaded CdO–TiO_2_ (CS-CT) and CdO–TiO_2_ (CT) nanocomposites under the irradiation of solar light. Additionally, the cell viability of the synthesized nanocomposites was carried out in HeLa cell lines under different concentrations, light doses and incubation periods using an LED light source. Compared to the CS-CT and CT nanocomposites, an efficient photodynamic activity was achieved in the case of AS-CT hybrid nanocomposites. Actually, the end-use properties required for both processes in AS-CT nanocomposites appear similar due to the presence of organo sulphurus compounds.

## Introduction

Nanotechnology has been supporting considerably for the development, even revolutionize various industries and technologies: transportation, medicine, food safety, energy, and environment, etc. It also been a boon in the field of environmental and medicine for over a decade. Photocatalysis based on nanomaterials is one of the most promising methods for environmental remediation. In this method, depending on the energy band gap, the nanomaterials are induced by different wavelengths of UV, visible, and NIR light^1^. The activated nanomaterials degrade the various chemical and microbiological pollutants in water by a photocatalytic redox mechanism.

In the domain of medicine, nanomaterials have an excellent potential for the treatment and diagnosis of cancer. Photodynamic Therapy (PDT) is a budding modality for cancer treatment favoring the interaction between photosensitizers (PS) and light to initiate cell death^2^. In PDT, the light activates the PS, not directly reacting with cells, but it transfers the triplet state energy to adjacent oxygen to generate a reactive singlet oxygen species that lead to the cytotoxic reactions^3,4^. Currently, many studies have addressed the feasibility of semiconductor nanoparticles (NPs), such as TiO_2_^5,6^, CdS^7,8^, Fe_2_O_3_^9–11^, ZnO,^12–14^ etc. in photocatalysis and PDT.

Semiconductor metal oxides offer an extensive interest owing to their vast potential applications. On this account, semiconductor NPs became extensively used in photocatalysis and cancer diagnosis. Among all the semiconductor oxides, Titanium dioxide (TiO_2_) has gained much interest in the environment and medicine. One of the applications of TiO_2_ semiconductor photocatalysts is the photokilling behaviour by the photoexcited TiO_2_ that could be applied in the biomedical field, particularly in cancer treatment^15^. During photocatalysis, the generation of holes and electrons have redox properties that cause various photocatalytic reactions^16,17^. In PDT, the presence of oxygen in the human body reacts with conduction band electrons of TiO_2_ generates reactive oxygen species (ROS), which damages the cancerous cell’s structure. The photokilling behaviour of TiO_2_ is feasible to use in PDT as a PS. However, TiO_2_ lacks its efficacy because of the low quantum yield and high band gap. Moreover, TiO_2_ has the disadvantage of being active only under the UV light region, which could decrease the photocatalytic activity and also, UV light could not penetrate deeply into the biological tissues. To overcome these difficulties, certain metal oxides are doped to reduce the band gap, thereby enhancing the photocatalytic and photodynamic activity^18^.

Dopants are metal oxides/metal ions that are added in small quantities (< 10%). Among the various toxic semiconductor NPs, Cadmium Oxide (CdO) is of the lowest toxicity^19,20^. Hence, CdO is considered a chemically compatible nanomaterial with the human body^21,22^. A heavy metal such as cadmium could eradicate tumor cells in the body even at low concentrations^23–25^. From this perspective, the therapy based on CdO NPs has been developed for nano-based treatments. The characteristics of TiO_2_ were strongly improved by doping with CdO. CdO, an n-type semiconductor with a band gap (2.18 eV) and a melting point of 1500 °C^26^. Dhatshanamurthi and his research team synthesized CdO loaded TiO_2_ and used it as a photocatalyst for degrading orange-4 dye. CdO–TiO_2_ possesses excellent photocatalytic activity than bare TiO_2_ under UV-A light^27^. In addition to the photocatalytic activity, first-principle calculations were used to determine the magnetic behaviour of Cd-doped TiO_2_ and found that potent ferromagnetism could be achieved by Cd doping with TiO_2_ in the concentration of 12.5%^28^. CdO nanomaterials are extensively involved in biomedical applications such as drug delivery, eradicating cancer cells and improving cancer cells' sensitivity for imaging and accurate observance^29–32^. Additionally, very few studies have been devoted to CdO doped TiO_2_ nanocomposites-based PDT for cancer treatment.

In the present scenario, biomaterials-based hybrid nanomaterials have acquired more interest in environmental and biomedical applications. The hybrid nanomaterials based on chitosan and garlic has been developed for various applications. Chitosan (CS), a chitin derivative and the most abundant natural polymer, otherwise called poly (1,4), b-D glucopyranosamine^33,34^. Recently, several research findings have been reported based on CS biopolymer^35–37^ and the results show that the production of new inorganic/organic nanomaterials with effective photocatalytic behaviour due to the immobilization of semiconductors onto CS. CS increases the dissolution rate of drugs with less solubility, targeting of drug and improves the drug absorption^38^. Due to its exceptional properties it also been applied in various biological applications such as analgesic, antimicrobial, hypocholesterolemic, hemostatic, and antioxidant activity^39–41^. The PDT associated with nanocarriers of CS are considered as one of the mechanisms for cancer treatment. This approach increases the PS’s specificity for targeting tumors, the solubility of photosensitive molecules and decreasing cytotoxicity^42,43^. Garlic *(Allium Sativum)(*AS), an herb that contains around 17 amino acids, 33 sulphurus compounds, and certain enzymes such as alliinase, peroxidases, myrosinase etc^44^. The loading of garlic with TiO_2_ and WO_3_–TiO_2_ hybrid photocatalytic materials notably enhanced the photocatalytic effect in the range of visible light^45,46^. Very few works reported that TiO_2_ NPs with optimized garlic loading possess outstanding activity against cancer and microbial than the bare TiO_2_ NPs. Indeed, the low price and the ease of synthesis constitute significant advantages of this material over the inorganic oxides.

**Scheme 1:**
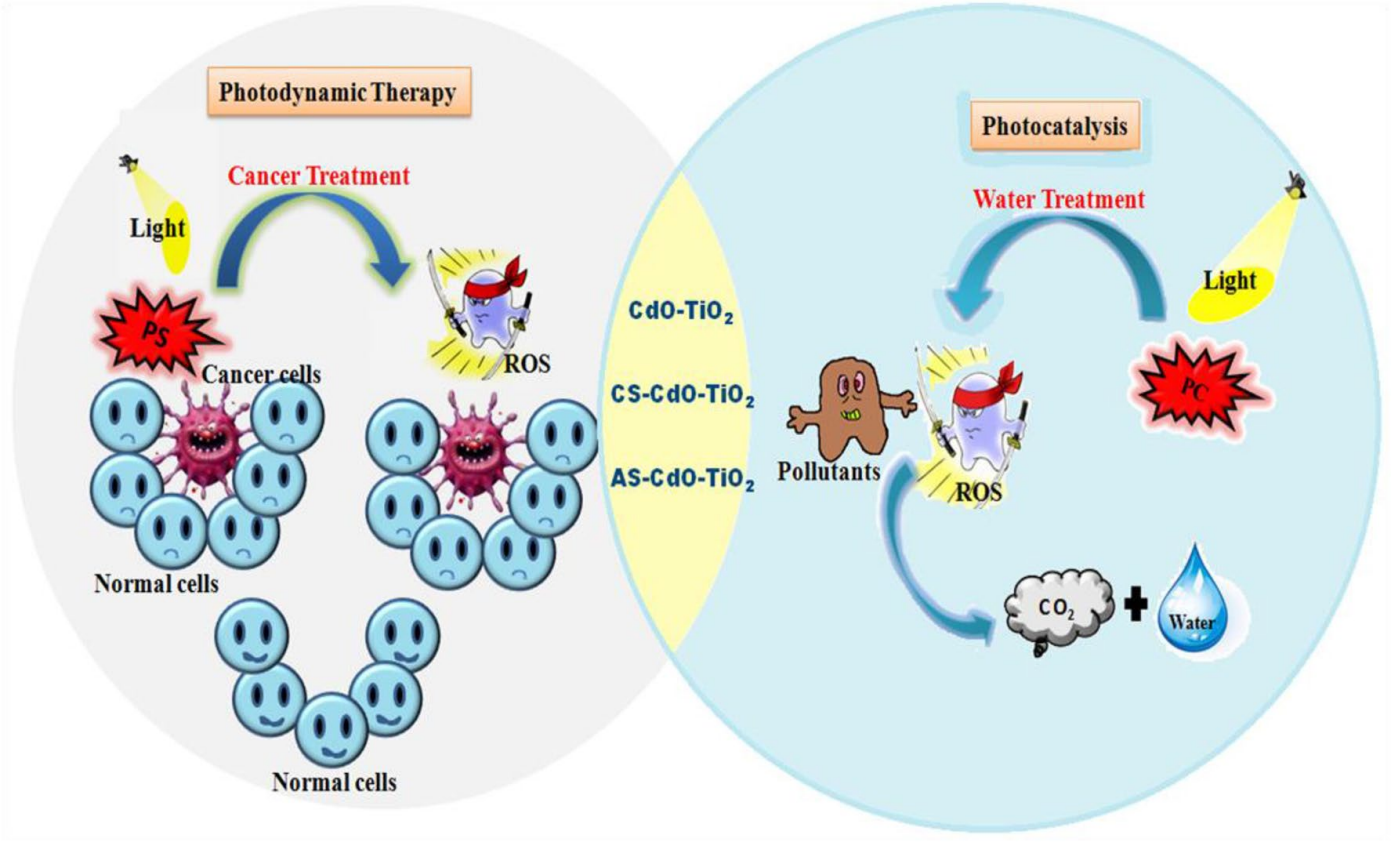
Illustration of the aim of the work.

To this aim, CdO doped TiO_2_ (CT), chitosan-loaded CdO doped TiO_2_ (CS-CT), and garlic-loaded CdO doped TiO_2_ (AS-CT) have been successfully synthesized. The photocatalytic activity of synthesized nanocomposites has been investigated against organic pollutants such as MB, MO and Rh-B under solar light. Additionally, the photodynamic activity of these materials has been investigated in the HeLa cell lines under the irradiation of an LED light source. Scheme 1 illustrates the objective of the work and application of synthesized bionanomaterials towards photocatalysis and PDT. Nonetheless, no previous attempts have been made to develop CS-CT and AS-CT hybrid NPs for photocatalytic and photodynamic activity.

## Result and discussion

### Characterization

**Figure 1:**
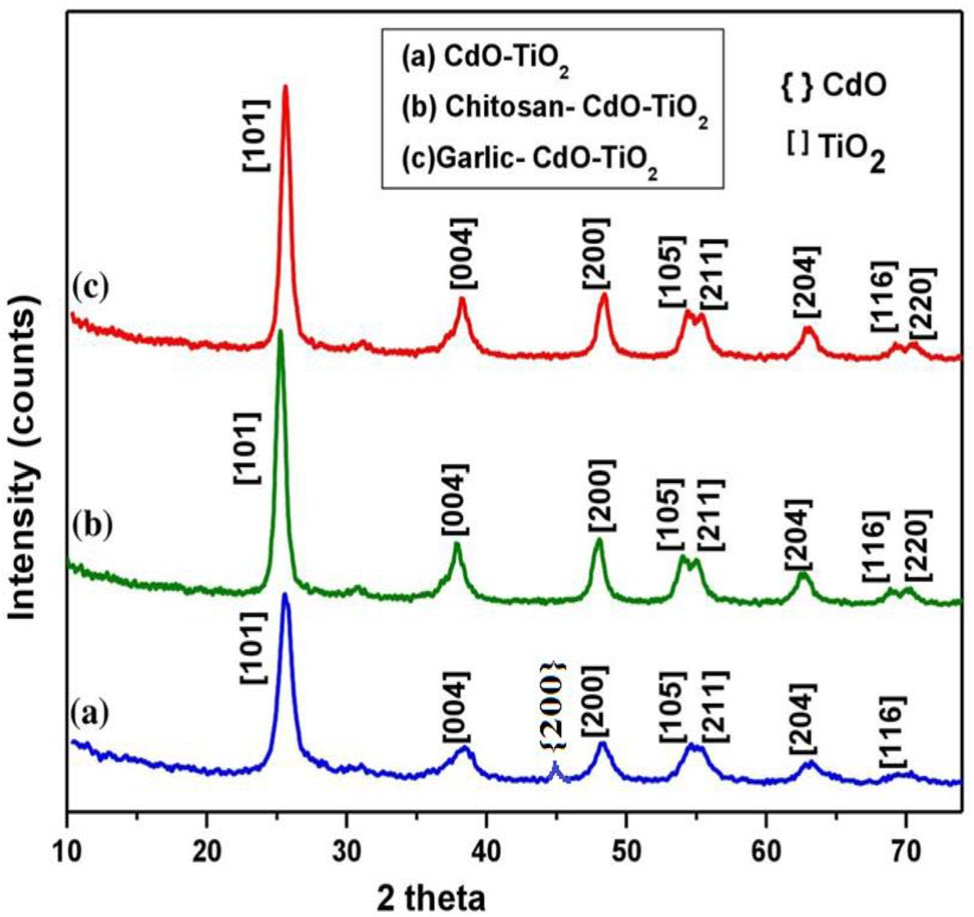
XRD of (**a**) CT (**b**) CS-CT and (**c**) AS-CT nanocomposites.

The XRD patterns of CT, CS-CT, and AS-CT nanocomposites were displayed in Fig. 1a–c. The peaks give information about anatase TiO_2_ and cubic face-centered CdO. At angle 2θ, intense peaks appear at 25.356°, 37.847°, 48.145°, 53.974°, 55.186°, 62.242°, 68.879° and 70.110° corresponding to the (101), (004), (200), (105), (211), (213), (116) and (220) planes have the best fit according to the standard JCPDS card No. 89–4921. The presence of peak at 2θ = 45.211° (Fig. 1a) corresponding to cubic CdO (05–0640) and parameters are: Cubic, face-centered, a = 4.689 Å, b = 4.689 Å and c = 4.689 Å, α = 90°, β = 90°, γ = 90°. The average crystallite size for CT, CS-CT, and AS-CT hybrid nanocomposites are 15, 11, and 8 nm, respectively. The sharp peak present in the XRD pattern indicates a highly crystalline structure and absence of impurity.

**Figure 2:**
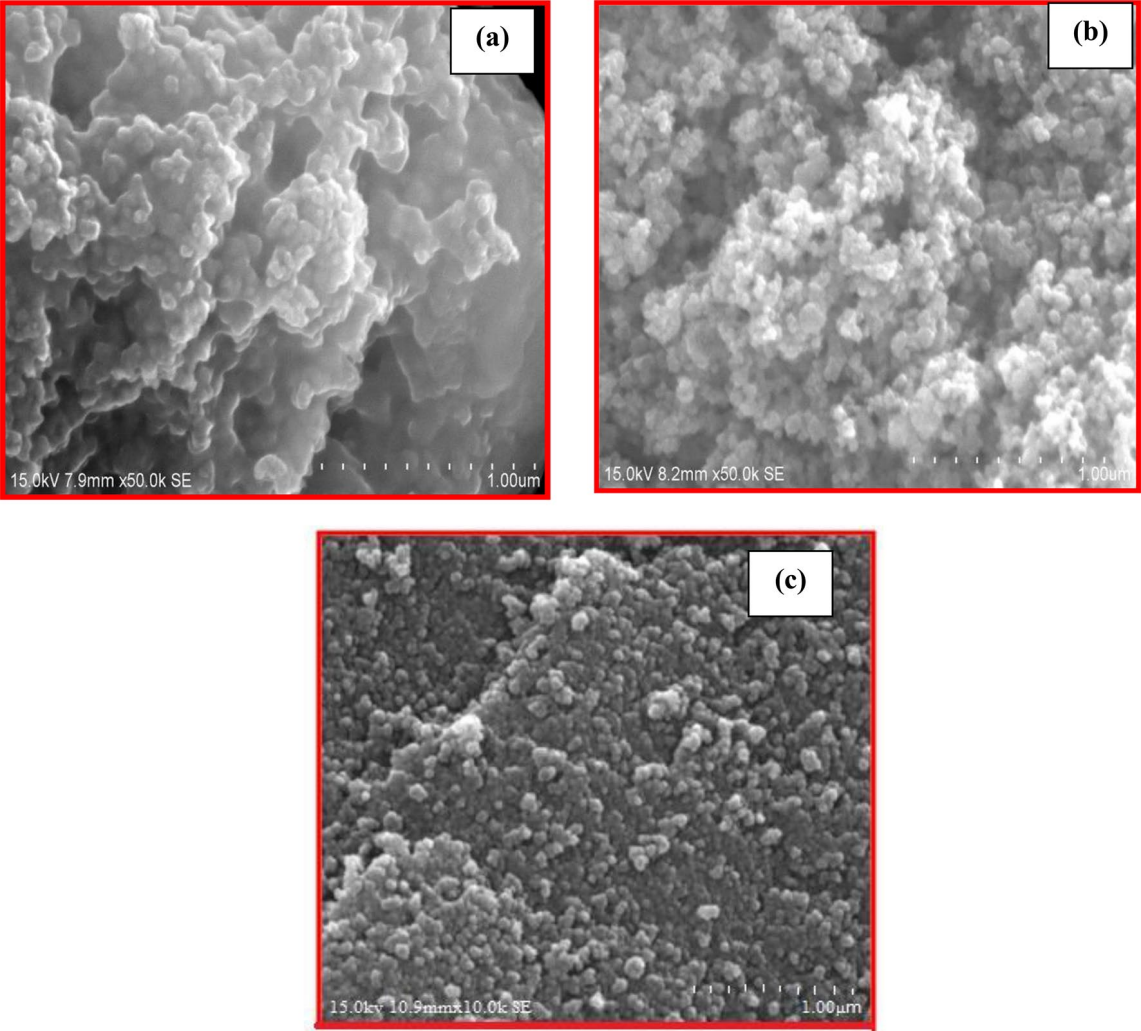
FE-SEM picture of (**a**) CT (**b**) CS-CT (**c**) AS-CT nanocomposites.

The effect of the catalyst relies on the morphological behaviour of the sample. The surface morphology of the synthesized nanocomposites was determined from FE-SEM analysis. The micrographs of CT, CS-CT and AS-CT hybrid nanocomposites were displayed in Fig. 2a–c. Compared with FE-SEM images of CT, the images of CS-CT and AS-CT (Fig. 2b,c) displays that the NPs are uniform and spherical in shape. The results highlight that the NPs appear spherically shaped with some irregularities. The particle size varies from 16 to 45 nm with an average crystal size of 8–15 nm.

**Figure 3:**
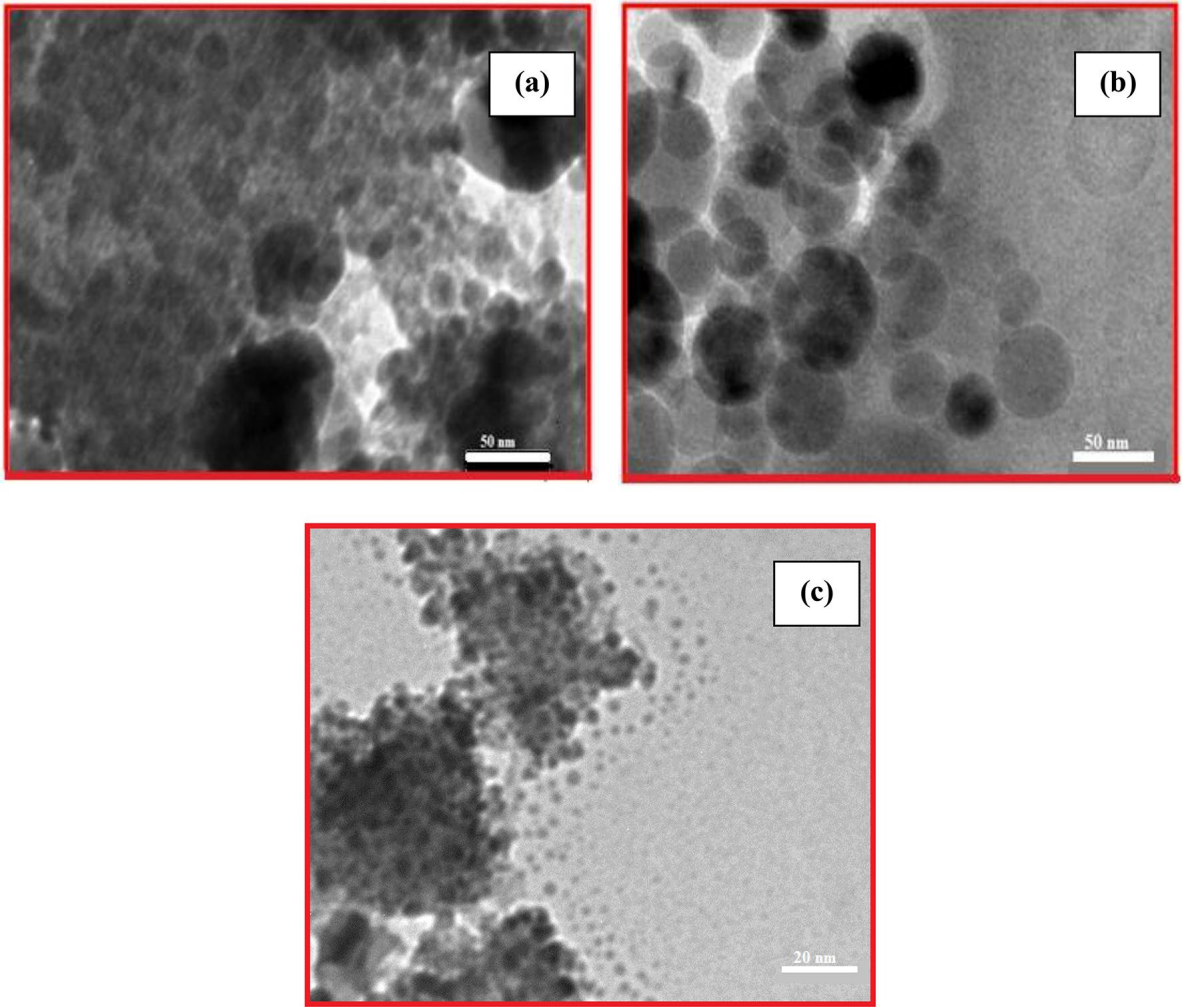
TEM picture of (**a**) CT (**b**) CS-CT and (**c**) AS-CT nanocomposites.

The TEM image of nanocomposites was presented in Fig. 3a–c. The present TEM images were undoubtedly exposed to FE-SEM reflection. Transmission electron microscope (TEM) images in Fig. 3a–c give close view of nanocomposites; there is no significant difference in the morphologies, which is almost spherical in shapes. However, there is a slight difference in particle size for all three synthesized nanomaterials but fall within the range of 16–45. This is about twice to thrice that of the average crystal size obtained from XRD and the nanocomposites are polycrystalline in nature. This result indicates that different sizes of nanocomposites might be obtained by using biomaterials.

**Figure 4:**
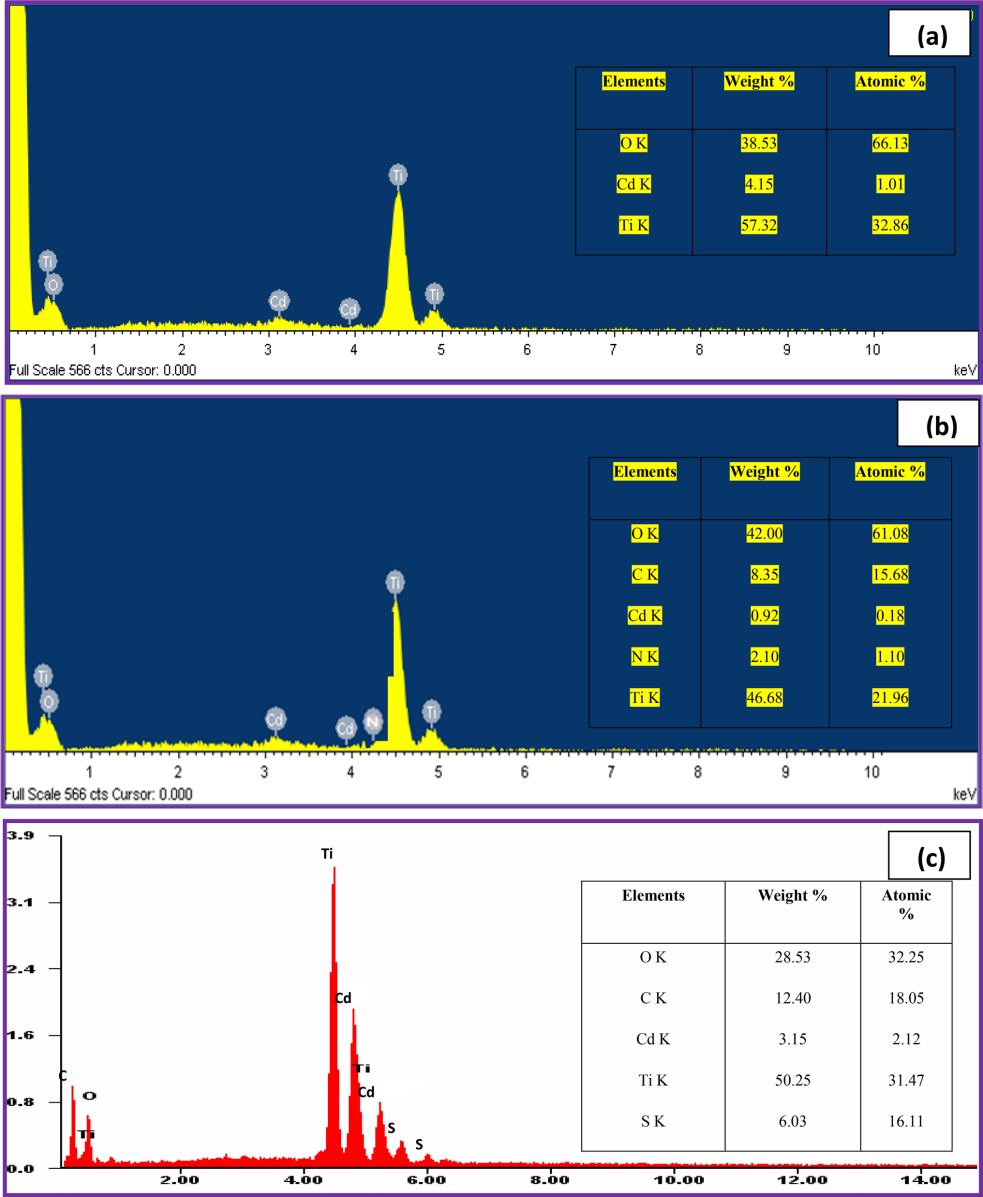
EDX of (**a**) CT (**b**) CS-CT and (**c**) AS-CT nanocomposites.

The elemental composition was detected by using the EDX technique. Figure 4a–c displays the EDX analysis of CT, CS-CT, and AS-CT nanocomposites. The results proved the existence of all the elements including, Ti, O, and Cd from TiO_2_ and CdO, in the synthesized nanocomposites. Similarly, presence of N and S along with Ti, O and Cd in CS-CT and AS-CT nanocomposites confirms the existence of chitosan and garlic in the bionanomaterials. In addition, the presence of O is associated with the oxygen in TiO_2_ lattice as well as in the surface –OH groups, and the Ti and O values are not even with the actual elemental composition of TiO_2._ The absolute amount of the elements present in the materials was not determined by using the EDX analysis, but the presence of specific elements can be determined^47,48^.

**Figure 5:**
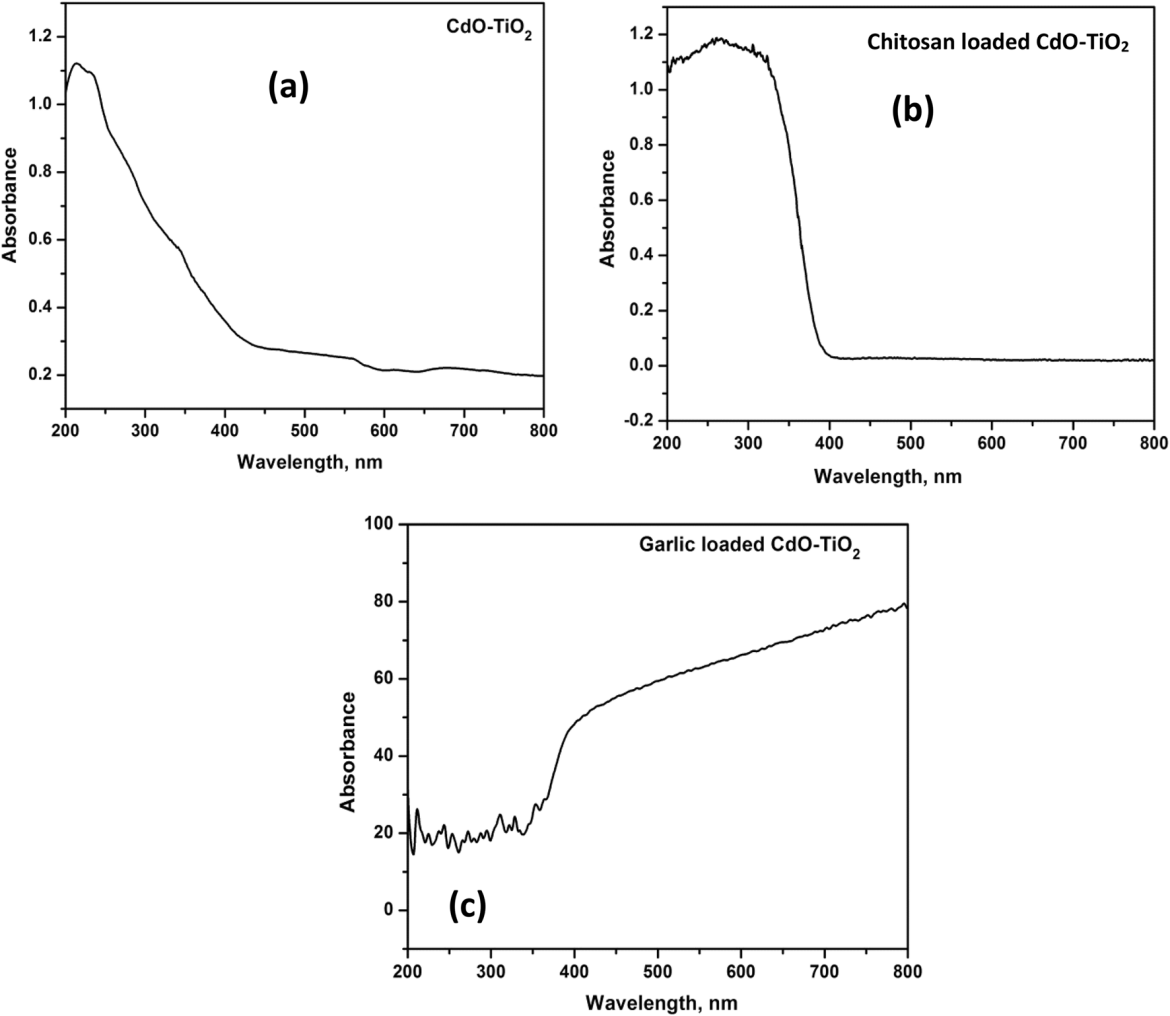
DRS of (**a**) CT (**b**) CS-CT and (**c**) AS-CT nanocomposites.

The visible light activity and the band gap of the synthesized CT, CS-CT, and AS-CT nanocomposites were studied by using UV-DRS. As shown in Fig. 5a–c, the light absorption characteristics of CT were also modified by loading of biomaterials. Figure 5a–c indicate that loading of biomaterial with CT nanocomposites had increased the absorbance from UV to the visible-light region, improving the photocatalytic and photodynamic behaviour of the nanocomposites. DRS of CdO-TiO_2_ and garlic-loaded CdO–TiO_2_ nanocomposites show absorption in the visible region of 500–600 nm. Chitosan-loaded CdO–TiO_2_ nanocomposites show absorption in the UV region. In the case of garlic-loaded CdO–TiO_2_ calcined at 450 and 700 °C, the absorption edge was observed in the visible region of the solar spectrum, that represents the catalyst excitation efficiently exploits more photons. This kind of absorption explained the substitution of titanium lattice by S^6+^, a newly isolated band formed above the valence band of TiO_2_ VB and the band gap narrowed consequently^49^. Furthermore, the results suggest that loading significantly promotes the band gap to red-shift, which eases the electron excitation from the VB to the CB that results in higher photocatalytic and photodynamic activity.

**Figure 6:**
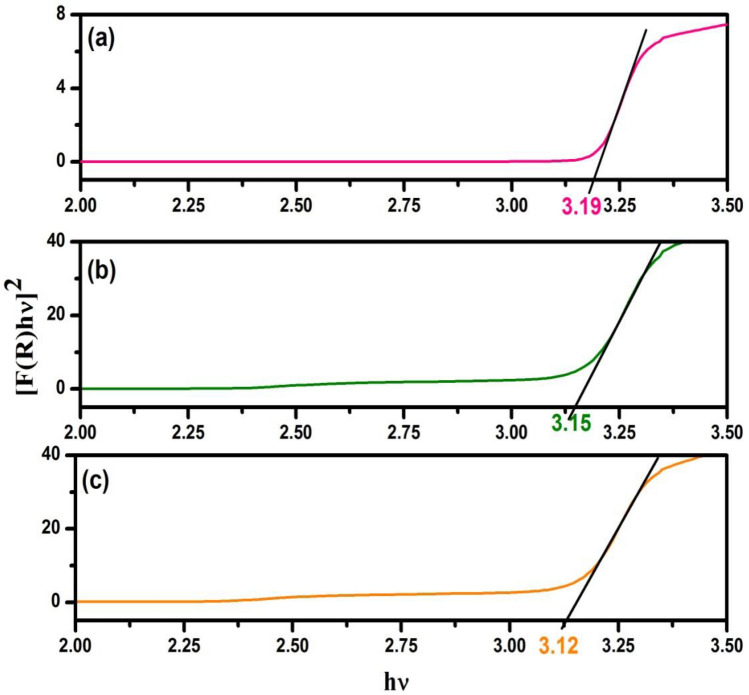
Direct band gap of the (**a**) CT (**b**) CS-CT and (**c**) AS-CT nanocomposites.

In the interest of predicting the type of band-to-band transitions in the synthesized nanocomposites, the absorbance data of the DRS was plotted into the direct band gap transitions equation^50^. Figure 6a–c gives [F(R)hν]^2^ *Vs*. photon energy plot for direct transitions. From the recorded reflectance (R), the F(R) data were deduced by Kubelka–Munk algorithm application [F(R) = (1–R^2^)/2R]. F(R) depicts the sample’s absorptivity at a specific wavelength. From the modified Kubelka–Munk algorithm plot, the corresponding wavelengths (λ_**g**_) and the absorption edges (*E*_**g**_) of the nanocomposites were determined.

**Figure 7:**
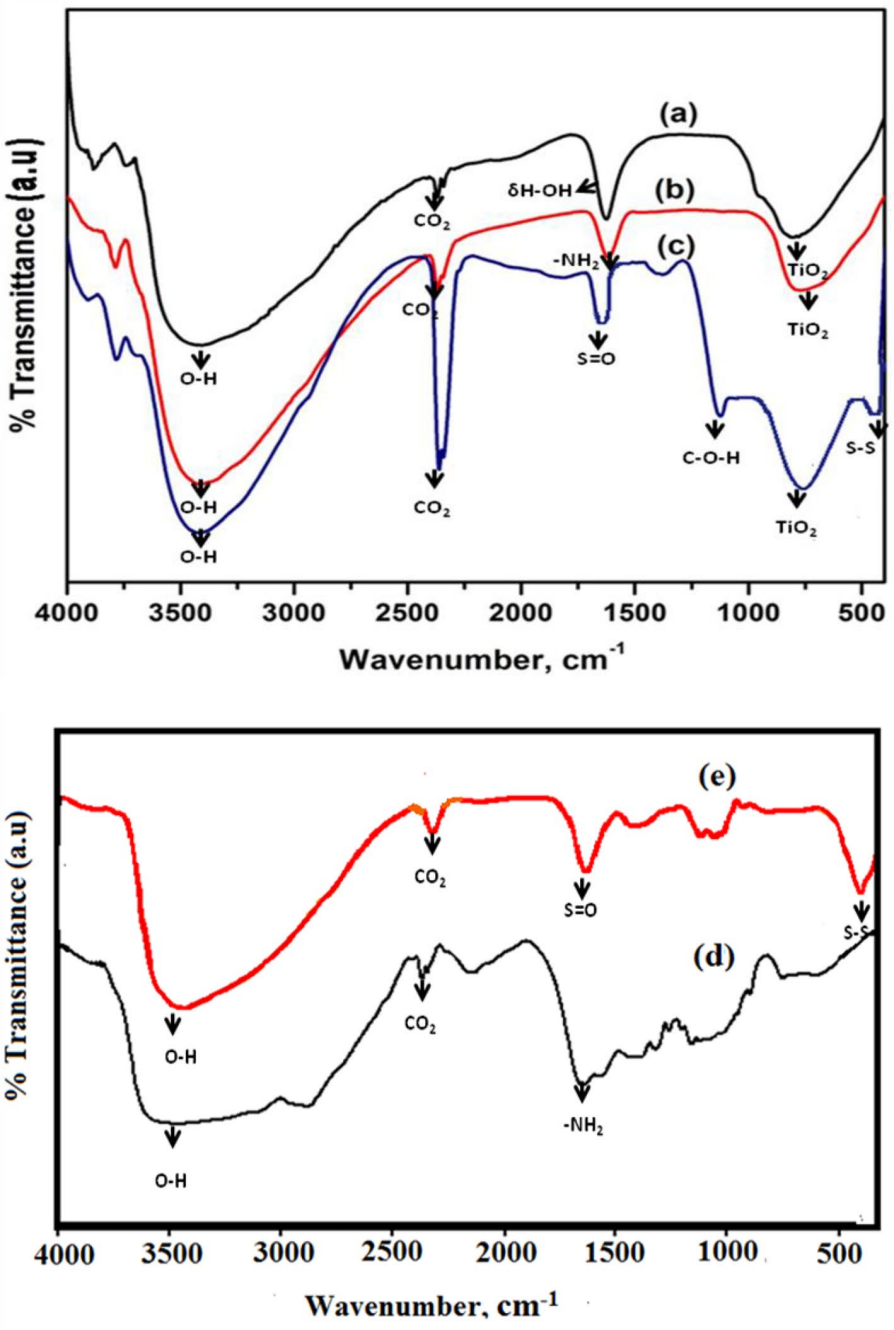
FT-IR of (**a**) CT (**b**) CS-CT (**c**) AS-CT nanocomposites (**d**) chitosan and (**e**) garlic.

The interaction in metal oxides and biomaterials doped TiO_2_ NPs was studied using FT-IR. The FT-IR analysis of the CT, CS-CT, AS-CT nanocomposites, chitosan and garlic extract were displayed in Fig. 7a–e. From the observance of Fig. 7a, a broad and strong transmittance band at 3400 cm^−1^ which is attributed to the O–H stretching vibration of TiO_2_ NPs. The peak at 2300 cm^−1^ corresponds to atmospheric CO_2_ vibrations, and a peak at 1630 cm^−1^ represents water deformation (δH-OH). A band in the range of 650 and 800 cm^−1^ corresponds to TiO_2_ different vibrational modes. In CS-CT hybrid nanocomposites (Fig. 7b) show a peak around 3400 cm^−1^ and 1630 cm^−1^ that indicates hydroxyl (–OH) and amine (–NH_2_) groups which behave as reactive and coordination sites for the organic species adsorption. A band at 700 cm^−1^ and 2300 cm^−1^ attributes TiO_2_ and the atmospheric CO_2_ vibrations. The presence of amide or amine and OH groups along with metal oxides favoring the confirmation of the efficient dye removal by adapting the process of photodegradation-adsorption^51^. AS-CT hybrid nanocomposites (Fig. 7c) display a band around 3400 cm^−1^ indicating OH groups stretching vibrations. The observance of a band at around 460 cm^−1^ corresponds to the S–S stretching of sulphur and a band at 1130 cm^−1^ is attributed to the C–O–H bending vibration of the carboxylic (–C = O) identified in compounds of garlic extract. The other peak observed is 1630 cm^−1^ indicates the C = C stretching exhibits strong stretching vibration and matches the earlier reports. A minor shift in the band at 1636 cm^−1^ for garlic-loaded CdO-TiO_2_ corresponds to S = O. A band at 633 cm^−1^ corresponds to strong TiO_2_ stretching vibration that indicates O–-Ti–O; the broadening of the peak occurs when loaded with garlic. For comparison, the FT-IR spectra of chitosan and garlic also displayed in the Fig. 7d,e. The presence of a peak at 1630 cm^−1^ corresponds to amine (–NH_2_) groups in chitosan and the observance of a peak at 1636 cm^−1^ corresponds to S = O functional groups in garlic along with the other peaks supports and matches with the synthesized bionanomaterials.

**Figure 8:**
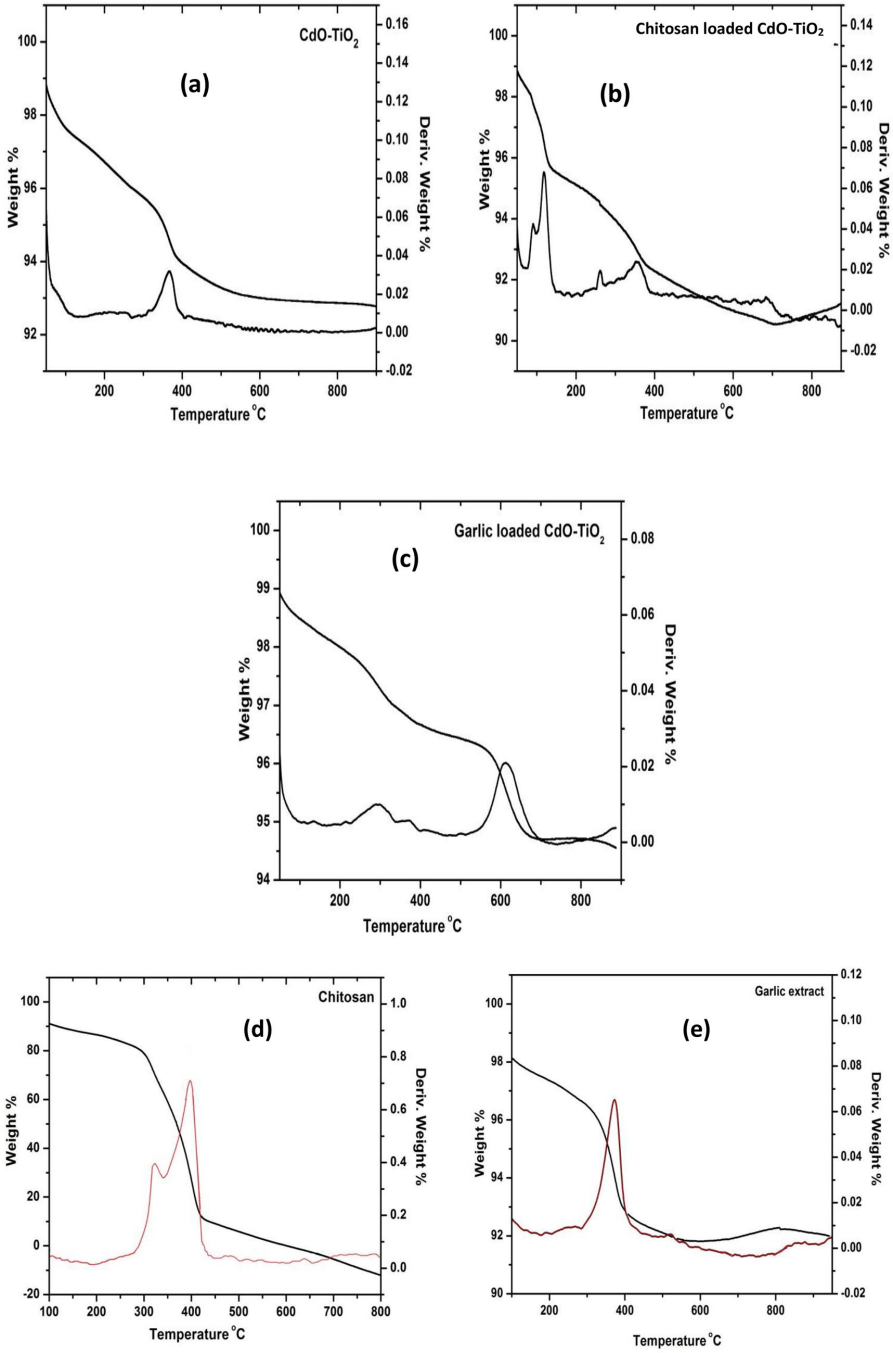
TG–DTA of (**a**) CT (**b**) CS-CT (**c**) AS-CT nanocomposites (**d**) Chitosan and (**e**) garlic.

Figure 8a–e gives the TG–DTA studies of the nanocomposites. From TG–DTA, the thermal behaviour of the nanomaterials was determined in the range of 50 and 800 °C at nitrogen atmosphere. The thermogram of CT nanocomposite (Fig. 8a) gives weight loss around 345 °C that represents the decomposition of the residual –OH groups. After that, there was no observance of peak since the compound remained intact. DTA graph shows a convexity appearance centered at 375 °C indicating the residual –OH decomposition. The thermo-gravimetric analysis of CS-CT (Fig. 8b) shows weight loss with two decompositions. The first weight loss that occurred at 115 °C is owing to the degradation of the chitosan polymer chain and the second weight loss at 354 °C is because of the crystallization of TiO_2_. The DTA of CS-CT nanocomposite reveals two prominent peaks. The first peak at 118 °C indicates the degradation of the chitosan polymer chain. The second peak at 354 °C may be corresponding to the template removal and TiO_2_ crystallization. Thermogravimetric analysis of AS-CT nanocomposite (Fig. 8c) shows two-weight losses with two decomposition steps. The weight loss below 300 °C is attributed to the decomposition of the residual –OH groups and the second weight loss at 620 °C is due to the phase transformation of TiO_2_. In DTA of AS-CT shows two convex appearances centered at 300 °C and 610 °C. The first exothermic peak is due to the removal of the residual OH group and the second weight loss is attributed to the phase transformation of TiO_2_. The TG–DTA of chitosan and garlic also displayed in the Fig. 8d,e, respectively. The TGA of chitosan shows two weight losses at 340 °C and 400 °C, first stage of weight loss was due to decomposition of the residual OH groups and the second stage of weight loss was attributed to the degradation of the polymer chain. In TGA of garlic extract, the major weight loss was observed at 400 °C that represents the main loss of natural extract. The noteworthy characteristic is that almost similar trends in mass losses were obtained up to 800 °C for all three synthesized nanocomposites.

### Photocatalytic activity

**Figure 9:**
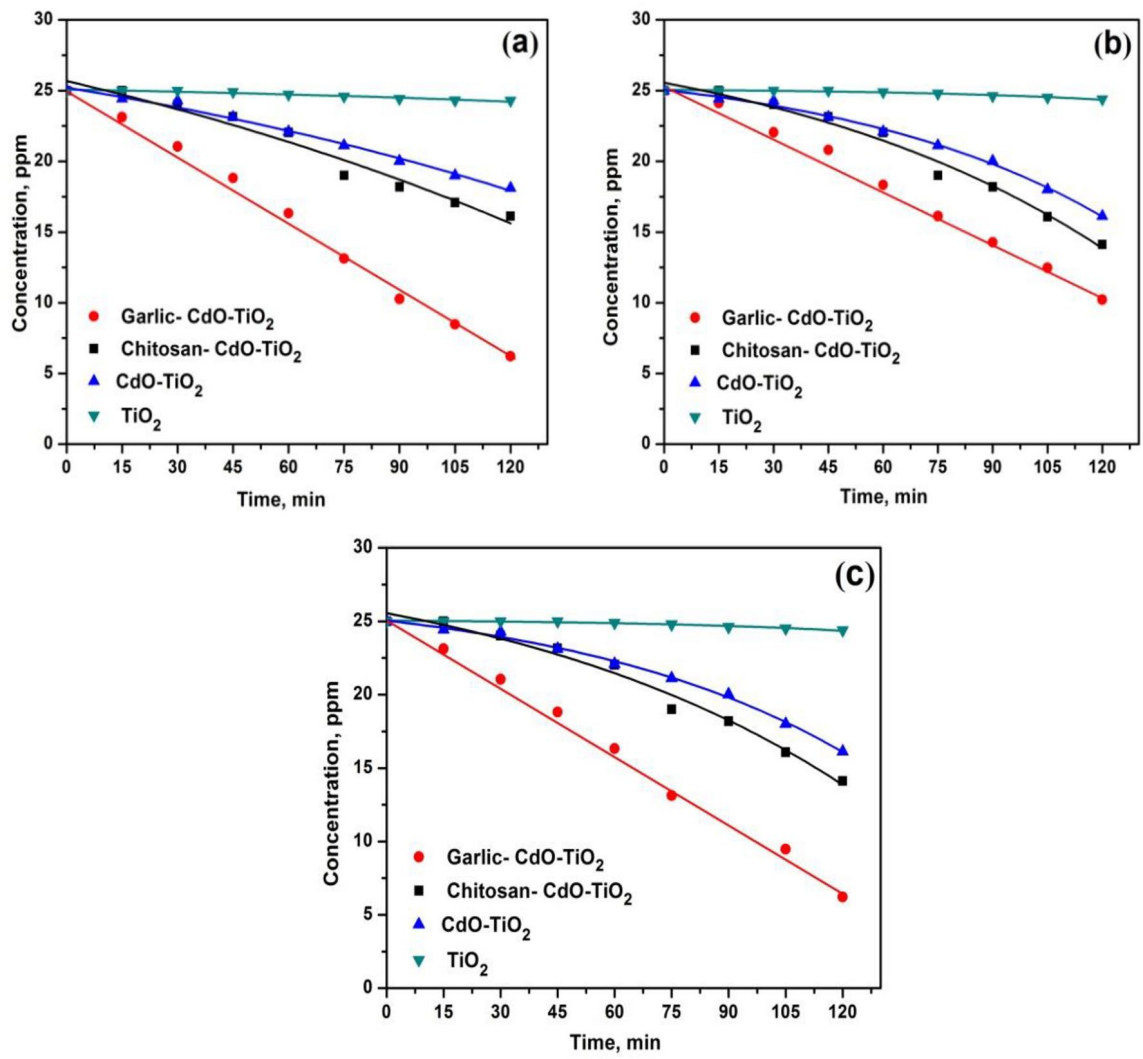
Solar light photodegradation profiles of the dyes (**a**) Rh-B (**b**) MB and (**c**) MO.

Figure 9a–c displays the photocatalytic behaviour of TiO_2_, CT, CS-CT_,_ and AS-CT nanocomposites under solar light have been analyzed using Rh-B, MB, and MO. Compared to other dyes (MB and MO), the degradation studies of Rh-B under the solar light irradiation show better activity for the synthesized nanocomposites AS-CT, CS-CT, and CT but frail for TiO_2_. The degradation order of Rh-B, MB and MO by the nanocomposites is AS-CT > CS-CT > CT > TiO_2._

The exhibited higher photocatalytic efficacy of AS-CT nanocomposite may be due to organic sulphurous compounds that possess a superior property of degradation^46^.

The second higher photocatalytic degradation of the dyes by CS-CT catalyst because of the anionic dye adsorption increased by a positively charged chitosan matrix surface. The amine groups present in the chitosan-based nanomaterials undergo protonation (formation of protonated amine), which could adsorb the dye molecules using various types of interaction mechanisms like chelation, electrostatic attraction, etc. It could have a higher adsorbent capacity to remove pollutants from the wastewater. It produces high active sites for complex formation with the attracted molecules, result in enhancement of the solar light photocatalytic efficiency^52^.

The CT catalyst also shows some favourable activity but not comparable with CS-CT and AS-CT nanocomposites. The significant photocatalytic effect of CT nanocomposite may be due to the dye-sensitized reaction. However, the catalytic deterioration of the dyes by TiO_2_ NPs is sluggish and there are no remarkable changes that could modify the degradation process.

The reason for enhanced photocatalytic activity of the biomaterials based hybrid nanomaterials is owing to (i) the high crystallization degree of doped/loaded anatase and stability that feeble the transfer of electron and subsequent reduction in the recombination of photo-generated holes, and/or (ii) the increase of vacancies of oxygen result in the doping or deformity lattice defects that attract the photoinduced electrons thereby suppress the e^−^  − h^+^ recombination^53–55^. Generally, the doped materials may deform the lattice of TiO_2_ and the substitution possible either Ti^4+^ or O^2−^. Thus, h^+^ in the valence band trapped by OH^−^ or the H_2_O adsorption produces the radical on the catalyst surface, whereas the photo-generated e^−^ in the conduction band reduces the adsorbed oxygen into ^**˙**^O_2_^­^ that enhances the catalytic activity^56^. In addition to that, the hole itself achieves the oxidation of the target pollutants effectively, which is adsorbed over the catalyst surface^57^.

The degradation behavior of the pollutants (Rh-B, MB, and MO) improved over the surface of the catalyst^58^ by the synergistic effect of generated radicals and holes but not processing in the bulk solution; the reason is the lifetime of the photo-generated radicals was short and inclined to the recombination^59^. Additionally, the occurrence of the enhancement of the dye degradation in the liquid phase has been made by the adding dopants of the slightly distorted lattice and presence of anatase phase with a high degree of crystallinity^60,61^.

### Recyclability and reusability

**Figure 10:**
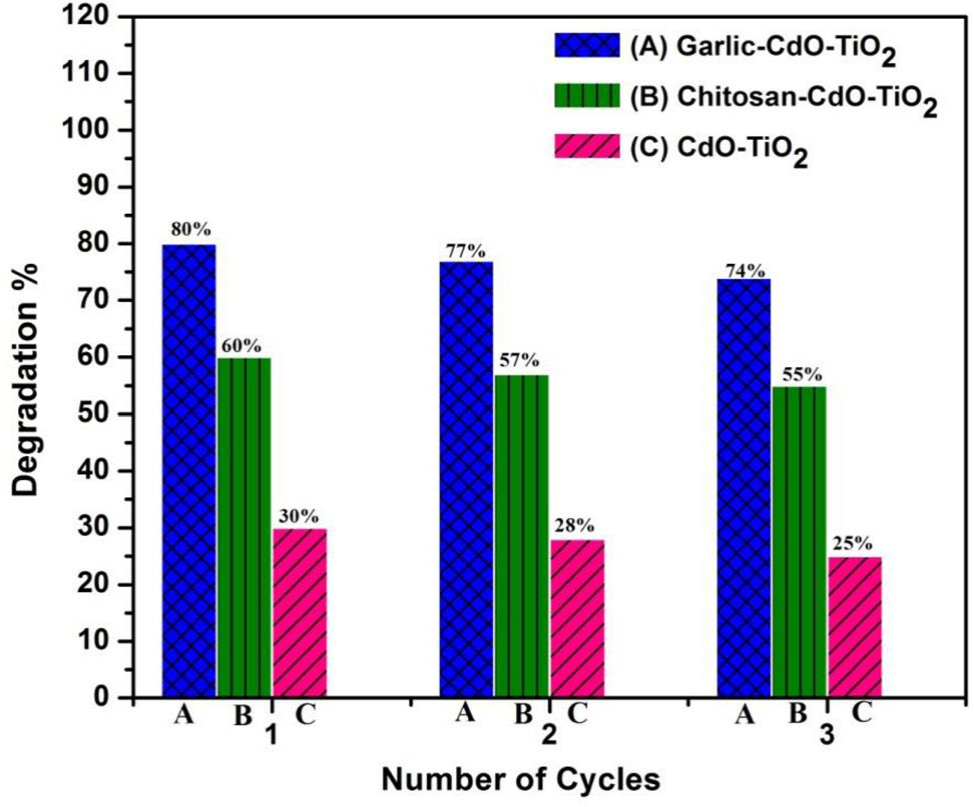
Recyclability of the synthesized nanocomposites.

The recyclability experiment was also carried out for the synthesized photocatalysts (Fig. 10). After each experiment, the photocatalysts were isolated from the reaction mixture, washed thrice by using absolute alcohol, oven-dried at 80 °C. The photocatalysts exhibit favorable reusability after 3 times of recycling. There was an observance of some extent of loss in catalytic activity after each reporting period. The fall in the photocatalytic activity/rate of photodegradation may be the result of loss in the amount of catalyst during the catalyst collection or weakening of the photocatalyst absorbing capacity.

### Cell viability

**Figure 11:**
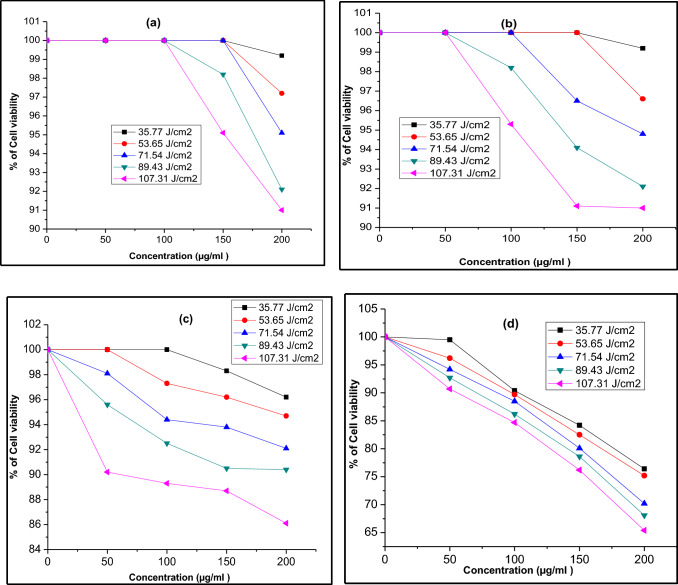
Cell viability of CT nanocomposites on HeLa cells after (**a**) 8 h, (**b**) 12 h, (**c**) 16 h and (**d**) 24 h of incubation.

**Figure 12:**
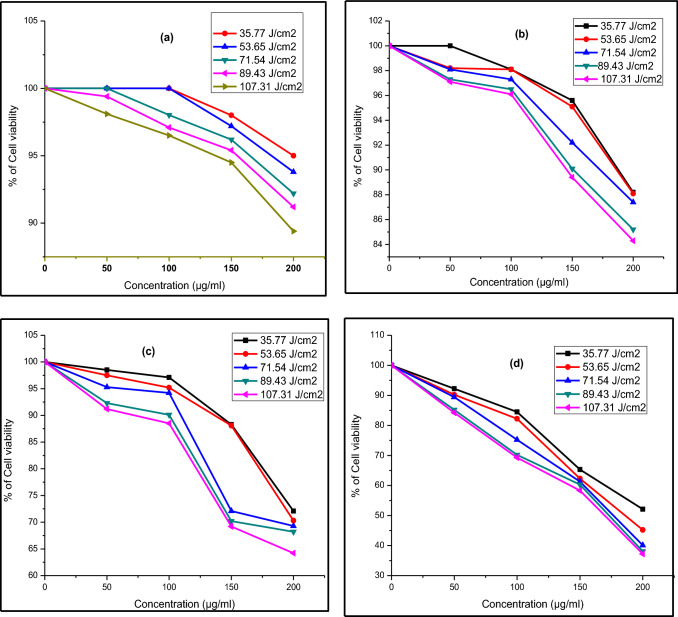
Cell viability of CS-CT nanocomposites on HeLa cells after (**a**) 8 h, (**b**) 12 h, (**c**) 16 h and 0 (**d**) 24 h of incubation.

**Figure 13:**
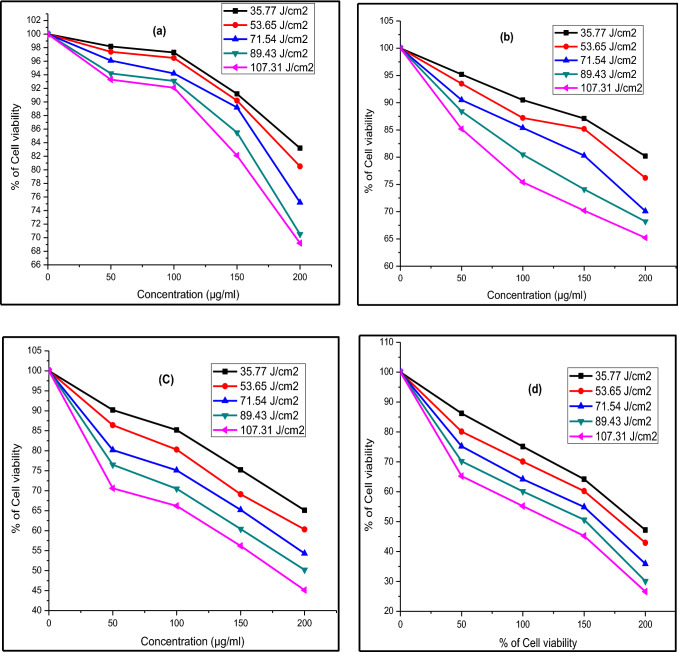
Cell viability of AS-CT nanocomposites on HeLa cells after (**a**) 8 h, (**b**) 12 h, (**c**) 16 h and (**d**) 24 h of incubation.

**Figure 14:**
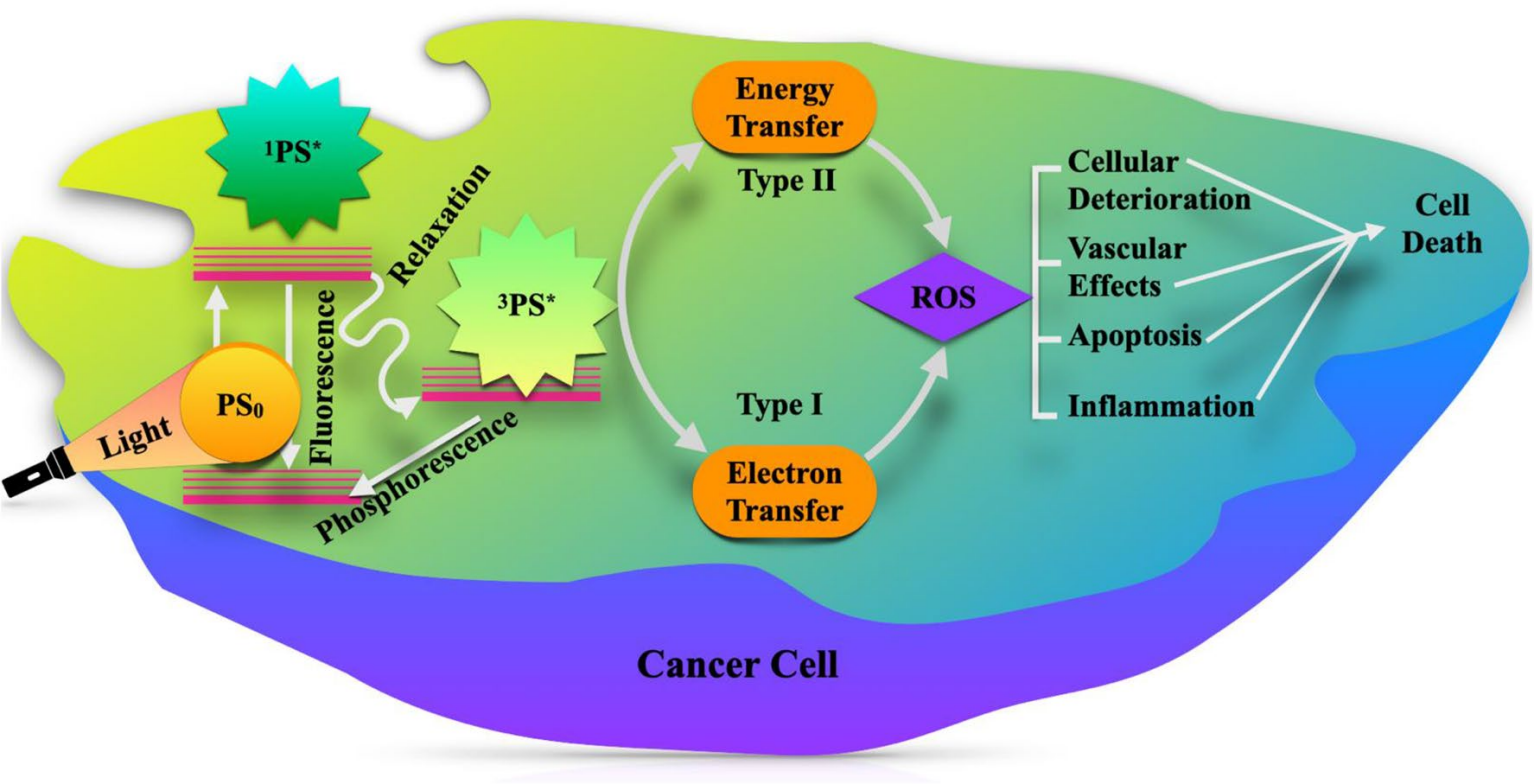
The plausible mechanism of PDT leading to cell death.

Figure 11, 12 and 13 display the difference in cell viability with various concentrations of nanocomposites and light doses. The plausible mechanism of PDT is provided in Fig. 14. The cell viability studies clearly explain the significant contribution of CT_,_ CS-CT, and AS-CT nanocomposites on the Hela cells and found to be both light dose, concentration as well as time-dependent. 100% indicates the presence of living cells in the control dish without nanocomposites. The cell viability rate decreases with the increasing concentration of nanocomposites and light dose.

The CT nanocomposites had no significant impact on the HeLa cells with increasing concentration, light dose and also incubation time (Fig. 11a–d). Considering the CS-CT effect (Fig. 12a–d), LC_50_ was found to be 184, 174, 171 and 167 µg/ml at the light dose 53.65, 71.54, 89.43 and 107.31 J/cm^2^ respectively after 24 h of incubation (Fig. 12d). Also, the clear observation reveals that further increase in the concentration of the dose above the LC_50_ level also had produced 60% of cell death at the different tested light doses except for the lowest dose at 36.77 J/cm^2^. Hence this is highly significant in considering the individual effect of CS-CT_._

Figure 13a–d shows cell viability of AS-CT nanocomposite on the Hela cells. Remarkably, AS-CT had shown increased anticancer activity when compared to the CS-CT. LC_50_ was found to be 189, 175, 159, 149 and 124 µg/ml for 53.65, 71.54, 89.43, 107.31 J/cm^2^ respectively for the AS-CT after 24 h of incubation (Fig. 13d). At the increased intensity of light dose tested at 89.43, 107.31 J/cm^2^, the concentration of anticancer activity slightly decreased to 159, 149 µg/ml for the AS-CT nanocomposite when compared to CS-CT nanocomposite, which was at 171 and 167 µg/ml, respectively. After 24 h of incubation, AS-CT nanocomposite shows a remarkable decrease in cell viability concerning the light dose and it was found to be 75% of cancer cells died for 107.31 J/cm^2^ of light dose at 200 µg/ml. It justifies that the garlic loading improve the anticancer effects by inhibiting Hela cells' growth. The efficiency of cell viability was progressively reduced with extending the incubation time. The times of incubation were optimized in order to confirm the time required for the maximum amount of cellular uptake by HeLa cells. The nanocomposites exhibit negligible dark cytotoxicity and significant light-triggered cytotoxicity. Meanwhile, the loading of garlic and chitosan on the surfaces of CdO-TiO_2_ nanocomposites facilitates the generation of ROS, enhancing the biological activity.

## Conclusions

Nowadays, biomaterials loaded with hybrid nanocomposites have been considered one of the foremost prevalent materials with potential applications in the environment and medicine. In this context, CT, CS-CT, and AS-CT hybrid nanocomposites were developed by simple sol–gel method. The structural, morphological and thermal properties of the nanocomposites were investigated through UV-DRS, XRD, HR-TEM, EDX, FE-SEM, TG–DTA and FT-IR analysis. The photocatalytic activity was evaluated by the degradation of Rh-B, MB and MO model pollutants under the solar light illumination. The AS-CT has an excellent photocatalytic activity towards the degradation of the pollutants over the CT, CS-CT nanocomposites.

Moreover, in vitro experiments on the HeLa cell line were performed to assess the PDT efficacy of those NPs under an LED light source and it demonstrated the efficient photodynamic activity of AS-CT than the CT and CS-CT nanocomposites. In both cases, the development of biomaterial-loaded NPs is devoted to enhancing photocatalytic and photodynamic efficiency. In particular, extending the activity towards the visible light domain and could enhance the generation of ROS. Most notably in AS-CT nanocomposites display better efficiency owing to the presence of organo sulphurus compounds. The synthesized nanocomposites exhibit low toxicity, high stability and good biocompatibility in vitro and in vivo. The information thus assembled could help to design new biomaterial-based nanocomposites specifically for photocatalytic and photodynamic applications in the future.

## Experimental section

### Materials

Cadmium Oxide (CdO), Titanium Tetra IsoPropoxide (TTIP), Tween-80, Ethanol and Isopropyl alcohol (IPA) were procured from Merck. Crab shells (Chitin) were purchased at Seafood Market, Kasimedu, Chennai, India. Methyl orange, Methylene blue, and Rhodamine B were supplied by S.D. Fine Chemicals, India.

### Synthesis of CdO-TiO_2_ (CT)

A suspension of 0.038 g of CdO and 20 mL of distilled ethanol was allowed to stir for 1 h for a homogeneous suspension. To the homogenous solution, 3 ml of tween-80 was added drop-wise and continued stirring for another 30 min. Followed by 3 mL of TTIP with 10 mL IPA was added drop-wise to the above mixture and allowed to stir for 2 h for obtaining gel. The gel obtained was separated and washed completely by using 1:1 aqueous ethanol and dried for 6 h at 120 ºC. The resultant material was calcined at 500 ºC for 3 h.

### Synthesis of chitosan loaded CdO-TiO_2_ (CS-CT)

The same procedure has been adopted up to the formation of a gel. The procedure for the CS synthesis from crab shells has been followed from the reported literature^62^. To the gel, CS solution (1 g of CS in 100 mL of 1% (v/v) acetic acid) was added and stirred for 1 h. The resultant product was filtered off and washed completely with 1:1 aqueous ethanol and dried for 6 h at 120 ºC. The samples were calcined at 500 ºC for 3 h by a Muffle furnace.

### Synthesis of garlic loaded CdO-TiO_2_ (AS-CT)

The same procedure has been adopted up to the formation of a gel. Freshly crushed garlic cloves were made into tiny pieces and grounded finely with a required amount of water for getting the garlic extract. To the gel, 10 ml of as-prepared garlic extract was mixed on continuous stirring and the resultant solution was allowed for ageing (24 h) and then filtered, dried for 6 h at 120 °C and calcinated at 500 °C for 3 h to get the corresponding AS-CT nanocomposites.

The experiments done on plants are in accordance with international, national and/or institutional guidelines.

### Instrumentation

Bruker D2 Phaser Desktop X-ray Diffractometer (Cu Kα radiation (λ = 1.542 Å)) was used to analyze the XRD. FE-SEM was investigated using a DXS-10 ACKT scanning electron microscope with EDAX. TEM images were examined from the JEOL JEM-3010 microscope with 600 and 800 k times magnification operated at 300 keV. Shimadzu 2100 UV–Visible spectrophotometer was used to study the DRS-UV Visible spectra between 200 and 800 nm. FT-IR spectrum was investigated by using a Perkin Elmer RX1 using solid KBr pellets. WATERS SDT Q 600 TA instrument was used to analyze the TG–DTA of the synthesized nanocomposites.

### Photocatalytic activity

The required amount of MB, MO and Rh-B dye solutions were prepared with doubly distilled water. MB, MO and Rh-B exhibit absorption maximum of 663, 464 and 555 nm and the absorbance of each dye at various ppm (MB—5 ppm, MO—20 ppm, Rh-B—5 ppm) was measured to construct the calibration curve. From the measured absorbance, the concentration of the dyes before and after illumination was determined.

To the as-prepared dye solution (50 mL), a required quantity of photocatalyst was added and kept under an air atmosphere at a constant rate. The photocatalyst was isolated after the illumination and the dye was measured spectrophotometrically.

The solar light intensities were evaluated by using a New 200,000 Lux Digital Meter Light Luxmeter Meter Photometer with Footcandle FC and the intensity was 1200 X 100 ± 100 lx and almost identical during the experiments.

All the photocatalytic analysis was conducted under the same environment on sunny days between 12.00–2.30 p.m. and the reaction mixture (50 mL) was illuminated with sunlight. An open borosilicate glass beaker with a capacity of 50 mL, a height of 40 cm and a diameter 20 mm was taken as a reaction container and the irradiation was applied in an open-air condition. Dye solution (50 mL) containing the photocatalysts was continuously aired and mixed thoroughly. No solvent volatility was noticed during the illumination. Periodically, sample solution (3 mL) was collected and the catalyst was separated by centrifugation. The sample (1 mL) solution was accurately diluted and its absorbance was measured at 555, 663 and 464 nm to evaluate the dye Rh-B, MB and MO degradation, respectively.

### Photodynamic activity

Human cervical cancer cell line (HeLa) was collected from NCCS, Pune and cultured in a medium with fetal bovine serum (10%). During analysis, HeLa cells (2 × 10^4^ cells/ml) were placed into wells of 2.5 cm diameter containing medium and incubated at 37 °C with 95% air and 5% CO_2_. The different concentrations of the samples (50, 100, 150 and 200 μg/ml) were added into the wells. The HeLa cells without the sample were used as control and the cells with samples were incubated at 37 °C for 3 h in a 5% CO_2_ atmosphere and irradiated at various time intervals (10, 15, 20, 25 and 30 min) using an LED light source (250, 300 and 410 nm). The HeLa cells viability was estimated at various incubation periods (8, 12, 16 and 24 h) after treatment.

The measurement of cell viability plays a fundamental role in all forms of cell culture. Cell-based assays are used to study the direct cytotoxic effects of drugs. Among the cell viability assays, the MTT assay (3-[4,5-dimethylthiazol-2-yl]-2,5**-**diphenyl tetrazolium bromide) is one of the most prominent methods for studying the mitochondrial dehydrogenase activity in living cells for safety and easy to use. In this method, viable cells convert MTT into a purple-colored formazan crystal having an absorbance maximum of around 570 nm. Thus, the color formation provides a convenient and effective marker of only the viable cells. 10 μl of MTT was added to each well after 8, 12, 16 and 24 h of incubation. The obtained formazan crystals were dissolved in dimethyl sulfoxide (200 μl) and the absorbance intensity at 570 nm was determined^63–65^.

$$\% \, \;{\text{of}}\;{\text{ Cell}}\;{\text{ viability}} = \frac{{{\text{Absorbance}}\;{\text{ of }}\;{\text{the}}\;{\text{ irradiated }}\;{\text{sample}}}}{{{\text{Absorbance}}\;{\text{ of}}\;{\text{ the}}\;{\text{ Control}}}} \times 100$$
